# Figuring out fidelity: a worked example of the methods used to identify, critique and revise the essential elements of a contextualised intervention in health policy agencies

**DOI:** 10.1186/s13012-016-0378-6

**Published:** 2016-02-24

**Authors:** Abby Haynes, Sue Brennan, Sally Redman, Anna Williamson, Gisselle Gallego, Phyllis Butow, The CIPHER team

**Affiliations:** 1 Sax Institute, Level 13 Building 10, 235 Jones Street, Ultimo, NSW 2007 Australia; 2 School of Public Health, University of Sydney, Edward Ford Building (A27), Fisher Road, Sydney, NSW 2006 Australia; 3 Australasian Cochrane Centre, School of Public Health and Preventive Medicine, Monash University, The Alfred Centre, 99 Commercial Road, Melbourne, VIC 3004 Australia; 4 School of Medicine, University of Notre Dame, 160 Oxford Street, Darlinghurst, NSW 2010 Australia; 5 Faculty of Health Sciences, University of Sydney, 75 East Street, Lidcombe, NSW 2141 Australia; 6 Centre for Medical Psychology and Evidence-based Decision-making, Level 6, Chris O’Brien Lifehouse (C39Z), University of Sydney, Sydney, NSW 2006 Australia

**Keywords:** Fidelity, Essential elements, Flexibility, Process evaluation, Intervention theory

## Abstract

**Background:**

In this paper, we identify and respond to the fidelity assessment challenges posed by novel contextualised interventions (i.e. interventions that are informed by composite social and psychological theories and which incorporate standardised and flexible components in order to maximise effectiveness in complex settings).

We (a) describe the difficulties of, and propose a method for, identifying the essential elements of a contextualised intervention; (b) provide a worked example of an approach for critiquing the validity of putative essential elements; and (c) demonstrate how essential elements can be refined during a trial without compromising the fidelity assessment.

We used an exploratory test-and-refine process, drawing on empirical evidence from the process evaluation of *Supporting Policy In health with Research: an Intervention Trial* (SPIRIT). Mixed methods data was triangulated to identify, critique and revise how the intervention’s essential elements should be articulated and scored.

**Results:**

Over 50 provisional elements were refined to a final list of 20 and the scoring rationalised. Six (often overlapping) challenges to the validity of the essential elements were identified. They were (1) *redundant*—the element was not essential; (2) *poorly articulated*—unclear, too specific or not specific enough; (3) *infeasible*—it was not possible to implement the essential element as intended; (4) *ineffective*—the element did not effectively deliver the change principles; (5) *paradoxical*—counteracting vital goals or change principles; or (6) *absent or suboptimal*—additional or more effective ways of operationalising the theory were identified. We also identified potentially valuable ‘prohibited’ elements that could be used to help reduce threats to validity.

**Conclusions:**

We devised a method for critiquing the construct validity of our intervention’s essential elements and modifying how they were articulated and measured, while simultaneously using them as fidelity indicators. This process could be used or adapted for other contextualised interventions, taking evaluators closer to making theoretically and contextually sensitive decisions upon which to base fidelity assessments.

**Electronic supplementary material:**

The online version of this article (doi:10.1186/s13012-016-0378-6) contains supplementary material, which is available to authorized users.

## Background

The process evaluation literature frequently characterises interventions as a ‘black box’ meaning that little is known about how they function, including the hypotheses that underpin their design [1–3]. Process evaluation shines a light in this box by investigating ‘how and why’ questions about the intervention’s implementation, change mechanisms and contextual interactions [4].

Fidelity assessment is a fundamental part of process evaluation. Its purpose is to ascertain ‘*the degree to which an intervention or procedure is delivered as intended*’ ([5]: 407). This is achieved by operationalising the intervention theory and monitoring the consistency and congruence with which it is implemented [6–9]. In order to determine if the delivery was ‘as intended’ two areas of assessment should be considered: implementation fidelity and theoretical fidelity. *Implementation fidelity* tells us to what extent the intervention-as-delivered matched the intervention-as-planned. The assessment focuses on measurable or codifiable dimensions such as how intervention providers were recruited and trained, what proportions of targeted people were reached, the amount of exposure participants had to intervention activities (intervention intensity) and the consistency with which the intervention components were delivered in each setting [10]. This is a comparative enquiry that identifies variation between desired and actual activities, between participant sites and over the duration of the intervention. Implementation fidelity assessment is vital for understanding the intervention’s variation [9, 11], determining its feasibility [6, 12] and determining whether an ineffective intervention was due to poor implementation or flawed design [3, 12–15].

*Theoretical* fidelity tells us the extent to which the intervention-as-delivered was congruent with the intervention theory (the logic and hypotheses that underpin the intervention design [16–18]). This intervention theory is operationalised in the form of ‘essential elements’: manifestations of the theory—the ‘active ingredients’—which must be implemented if the intervention is to be effective [2, 6]. The assessment uses the intervention’s essential elements as indicators for a formative enquiry that makes judgements about the validity of the intervention design in practice. This helps us determine how the intervention worked or why it did not [17–19]. As the new UK Medical Research Council guidance for process evaluation states,

*It may never be possible to fully understand how variations in delivery affect outcomes, given that adaptations do not occur at random, and will be confounded by factors promoting or inhibiting intervention effects. A strong understanding of the theory of the intervention is a prerequisite for meaningful assessment of implementation, focused not just on the mechanics of delivery, but whether [the] intervention remained consistent with its underlying theory* ([4]: 41).

Ensuring theoretical fidelity is vital for assessing the program theory [14], predicting outcomes [9, 20, 21], translating and adapting interventions for other contexts [12, 19, 22], further developing the intervention’s evidence base [9, 23] and enabling ‘streamlining’ that may reduce burden and cost [6, 24]. In trials of complex interventions, fidelity assessment supports interpretation of intervention outcomes ensuring that observed effects (or lack thereof) can be linked to implementation of the intervention. More positive outcomes have been observed when interventions are delivered with high implementation and theoretical fidelity [9, 12, 18], including in flexible interventions providing that adaptations are locally and culturally appropriate and are congruent with the program theory [11, 25–28].

The concept of assessing fidelity as part of intervention evaluation originates from psychotherapeutic programs. The aim of fidelity assessment in this context is to ensure prescribed treatments are delivered with minimal variation [15, 21] and adhere to the behaviour-change theory that informed their design. This approach has proliferated within implementation science and is now used for a range of interventions designed to change professional practice in health care. There is increasing formalisation of the theory that underpins these interventions and their essential elements, leading to testable theoretical frameworks and taxonomies of standardised techniques that support replicability and evidence synthesis across studies, e.g. [29, 30].

However, this approach cannot be used for all intervention trials. Indeed, its proponents do not suggest that methods designed to assess the fidelity of ‘*clinical actions performed by healthcare workers in the process of delivering healthcare*’ [30] should necessarily be more widely applied [31]. Two aspects in particular pose problems for translation: (i) the focus on individual behavioural change and (ii) the specificity with which the theory is operationalised. The former is problematic because the best-developed methods of fidelity assessment identify essential elements from a taxonomy of techniques derived from individual behaviour-change theory [29, 32]. No equivalent exists for interventions informed by broader social science theories that target complex interactive, organisational and system level properties [10, 33, 34]. The latter is problematic because it is too restrictive for assessing the fidelity of flexible interventions designed to allow local adaptation in order to increase their relevance and applicability [35–37]. Nor does it capture how interventions respond reflexively to unique characteristics and unpredictable reactions in their settings [38]. This *fidelity*/*adaptation dilemma* [22] is particularly pertinent for interventions based on composite theory that are designed for dynamic real world systems in which it is necessary to balance standardisation of both form and content with responsivity to context. Indeed, resolving the fidelity/adaptation dilemma in these contextualised interventions is one of the most important challenges for evaluation [39]. (For clarity, we use the term *contextualised intervention* rather than *complex intervention* in this paper as complex interventions are most commonly defined in relation to structural design rather than their theoretical or contextual characteristics [40].)

A growing body of literature documenting the evaluation of contextualised large-scale interventions attempts to tackle the challenges of composite theory, flexibility and responsivity to context. These interventions include those informed by ecological, complexity, empowerment and realist perspectives, and those tailored by local providers or developed participatively, e.g. [35, 41–48]. However, while many studies link their intervention’s essential elements to theory, they seldom report sufficient detail for others to see how that theory was translated into specific intervention techniques (rather than other techniques or variants that might be equally well supported by the theory). Moreover, some assume prior knowledge of the form that the intervention and its underlying theory will ultimately take, failing to acknowledge that an intervention’s so-called *essential* elements may function as conditional elements: contingent on the interaction between intervention techniques, heterogeneous participants and contextual characteristics [49–52]. Consequently, the intervention designers may be obliged to make countless incremental adjustments to the techniques and the theory that underpins them while the trial is in progress; thus, ‘*By the end of the program, the designers’ operating theory may look quite different from the theory with which they started*’ [53]. Intervention studies targeted at community populations such as cultural groups often highlight the contingent validity of program theory and why it should be critiqued, (re)operationalised and potentially rejected, depending on local needs and conditions, e.g. [27, 48, 54], but this is often lacking in organisational level studies [51]. So few trials conducted in policy organisations have been reported that, currently, our knowledge of how intervention strategies may interact with variations in these environments is little more than speculative.

Despite widespread agreement that all intervention trials should document the extent to which their essential elements were delivered [6, 12, 36], no universal methodology exists for identifying or measuring essential elements [8–10, 55] and, for interventions with composite theory, there is sparse guidance for ensuring putative essential elements are valid indicators of the underpinning theory [9, 20, 38, 55]. So how should we determine which elements of an intervention are genuinely essential and which can be adapted without impairing effectiveness? Calls for greater attention to these questions are widespread, coming from multiple sectors in health [5, 6, 13, 17, 23, 38, 56, 57], education [19, 55, 58] and community development [11, 20, 35, 59].

### How are essential elements identified?

When based on previous studies, intervention designers can identify essential elements from analysis of earlier interventions or operationalise them using exemplary models that have established effectiveness [9, 10, 12]. Theoretically informed standardised behaviour-change techniques are in development, but these are currently limited to interventions founded on psychological theories [30]. When designing and evaluating novel contextualised interventions, designers can either articulate the essential elements themselves or consult with expert colleagues [8, 9, 19, 56]. Many evaluations tackle this post hoc, piecing together the essential elements via discussion with the designers and/or by reviewing intervention materials [12, 19, 55].

The design of interventions in trials is often founded on an amalgam of hypotheses that attempt to take account of inter-related theoretical, contextual and pragmatic factors. These include formal and substantive theories; hunches based on professional experience; and considerations such as study resources, demands on participants, existing practice and infrastructure constraints. The intervention’s essential elements are representations of these composite working hypotheses [55]. Thus, essential elements are not extant change agents waiting to be discovered; rather, they are ways of putting working theories into practice in particular circumstances, chosen as the ‘best bet’ from many potential candidates [7]. It is not surprising, therefore, that newly developed essential elements for all types of intervention need to be assessed in situ to determine the extent to which they capture and truly deliver the intervention theory in the context of messy real world delivery [17].

### How specific should essential elements be?

The degree to which essential elements are specified must align with the level of flexibility in the intervention design. Minimally specified essential elements are appropriate for highly flexible interventions because they can be interpreted for different contexts [34, 60, 61]. These essential elements tend to be expressed as principles, goals or functions (rather than specific techniques or formats) as these provide scope for diverse implementation strategies. Fidelity rests on the extent to which the resulting strategies align with the principles, goals and/or functions (see [59] for examples) [33, 62]. Equal emphasis should be placed on how discretionary elements were tailored and with what process effects [33, 59].

Where the intervention combines standardised and flexible components, an appropriate balance must be found. Essential elements that are too tightly specified oblige providers to adhere to prescriptive scripts and techniques which may be suboptimal or entirely inappropriate in different contexts and circumstances [27, 35, 62], whereas minimally specified essential elements may not provide sufficient concrete guidance for developing or monitoring the core intervention activities [21]. The specificity of essential elements is critical for defining what the intervention *is* and what it *is not*, including which elements are genuinely essential and which can be adapted [13, 55]. To date, the literature does not provide the detail needed to identify, or determine the specificity of, essential elements for contextualised interventions.

### Aims

In this paper, we identify and respond to the challenges of fidelity assessment in contextualised interventions using the Supporting Policy In health with Research: an Intervention Trial (SPIRIT) study as an example. SPIRIT is testing the effects of a suite of strategies designed to increase the capacity of health policy agencies to use research. SPIRIT recognises that policymaking is a messy subjective social process that takes place in complex open systems with myriad influences [63]. How research is used in policymaking is not fully understood [64], but it appears that different structures, pressures, relationships, values and events interact to shape its relevance, applicability and use, and that this flux cannot be controlled during interventions [22, 43, 64, 65]. Consequently, SPIRIT draws on diverse theories from social and political science, targets individual and system level capacities and, as Table 1 shows, attempts to balance standardisation with responsivity to context in its implementation and evaluation.

**Table 1:** The degree of flexibility in SPIRIT intervention components and subcomponents

Intervention components (fixed)	Subcomponents	Targeted participants	Degree of flexibility in form and content^a^
1. Audit, feedback and goal setting	a. Feedback forum	Senior leaders and other key managers, as determined by each agency	Partial: Tailored presentation based on agency’s audit data. Informal discussion shaped by participants’ interests.
	b. Intervention selection		
	c. Identification of other strategies		
	d. Mid-intervention feedback		
	e. SPIRIT newsletter	All agency staff involved in policy/program work	Partial: Tailored to each agency based on their audit data
2. Leadership program	a. Supporting organisational use of evidence	Senior leaders and other managers depending on size and configuration of agency	Partial: Standardised presentation determined in consultation with providers, but with agency-specific case examples. Discussion shaped by participants’ interests.
	b. Leading organisational change		
3. Organisational support for research	a. Quarterly email endorsement of SPIRIT from agency’s CEO	All agency staff involved in policy/program work	Partial: Proforma text that CEOs can adapt
	b. Access to WebCIPHER (an interactive research portal)		Limited: Web CIPHER is an online knowledge exchange community providing news, events, research, reviews and resources relevant to health policy.
	c. Resources for improving the agency’s use of research		None: Agencies were given the same generic resources.
4. Opportunity to test systems for accessing research and reviews (brokered services)	a. Brokered commission of: ­ a rapid systematic review OR ­ an evaluation plan OR ­ an analysis of linked data	Primary: Agency-selected staff who would benefit from experience commissioning a service. Secondary: all staff working in the topic area addressed by the commissioned product	Extensive: Standard brokerage processes are used but agencies choose the product, and specify the topic and how it should be approached to best meet their needs.
5. Research access	Three occasions of research access from two modes:	All policy/program staff working in the topic area covered by the forum	Extensive: Agencies choose the topic. They can nominate a particular provider and negotiate the form of the session. Providers shape the delivery details.
	a. Interactive forums with researchers AND/OR		
	b. Summary of systematic reviews	All policy/program staff working in the topic area covered by the review	Partial: Agencies choose the topic
6. Educational symposia for staff	a. Valuing research symposium	All agency staff involved in policy/program work	Limited: Agencies can nominate case examples
	b. Two symposia from: ­ Access to research ­ Appraising research ­ Evaluation ­ Working with researchers	All policy/program staff who might benefit from enhanced skills in the techniques covered	Partial: Agencies select symposia topics from the menu. They can tailor the focus, nominate case examples and providers, and negotiate the form of the session. Providers shape the delivery details.

^a^In all cases agencies had the scope to negotiate session dates, times, duration (between 1-2 hours) and attendance

Specifically we (a) describe the challenges of, and propose a method for, identifying the essential elements of a contextualised intervention (a semi-flexible, theoretically eclectic intervention designed for complex settings); (b) provide a worked example of an approach for critiquing the validity of putative essential elements; and (c) demonstrate how essential elements can be refined during a trial without compromising the fidelity assessment. We consider how this approach might complement current methods for identifying essential elements.

### Context for this study: SPIRIT

**Fig. 1:**
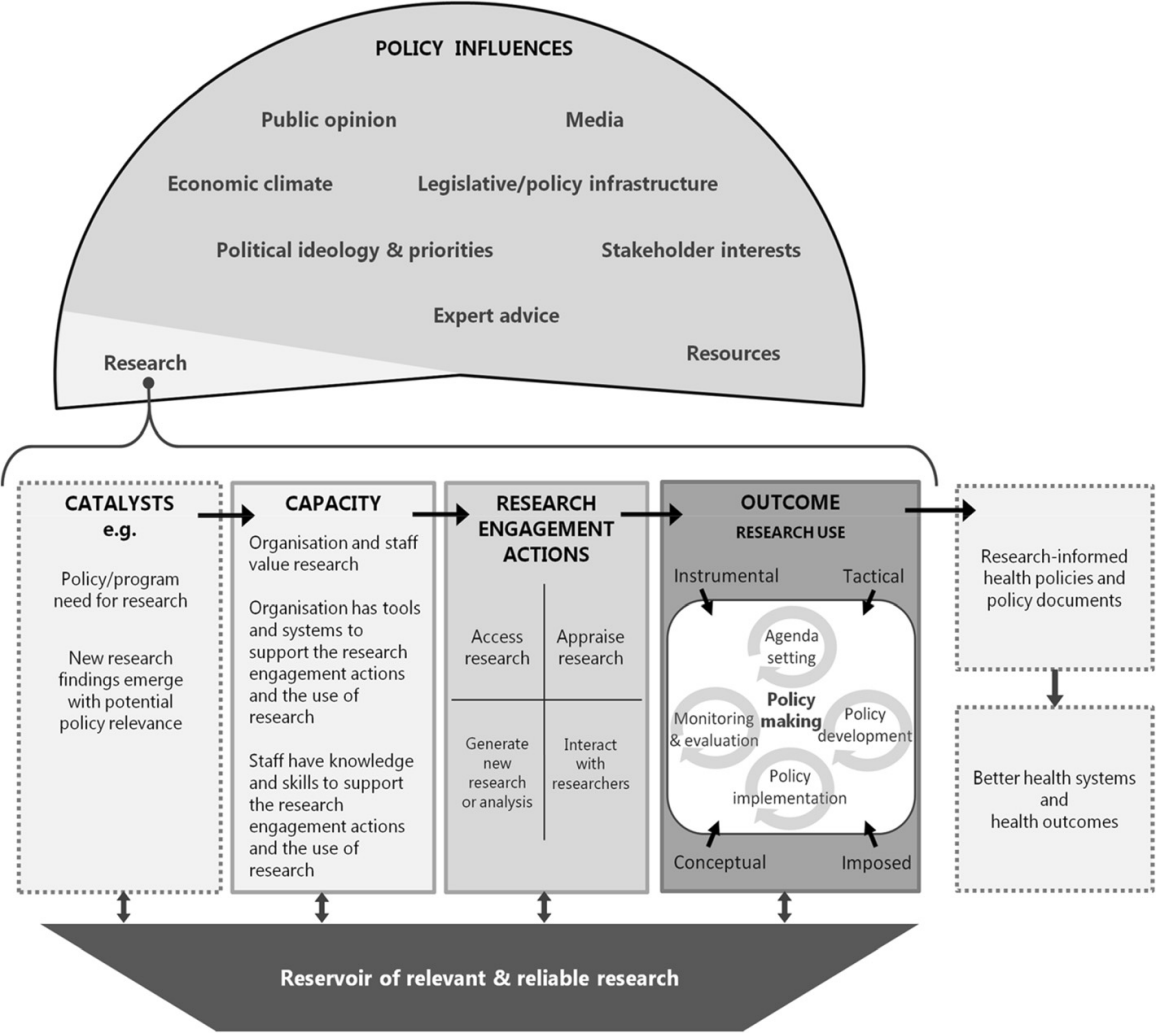
The SPIRIT action framework. From: Redman, S., Turner, T., Davies, H., Williamson, A., Haynes, A., Brennan, S., Green, S. (2015). The SPIRIT Action Framework: A structured approach to selecting and testing strategies to increase the use of research in policy. Soc Sci Med, 136-137, 147-155. doi:10.1016/j.socscimed.2015.05.009

Our fidelity assessment was developed and conducted as part of the process evaluation of Supporting Policy In health with Research: an Intervention Trial (SPIRIT). In this trial, six health policy and program agencies based in Sydney, Australia, participated in an intervention designed to increase the capacity of policymakers and program developers to use research in their work. SPIRIT was informed by cognitive behavioural theory, systems thinking, the literature on research utilisation, organisational change and adult learning theories. These were articulated in the form of the SPIRIT action framework (Fig. 1) and a list of change principles (Table 2) which, in turn, guided the intervention design and the goals and strategies of individual activities [63, 66].

**Table 2:** SPIRIT change principles

Systems framework	• Uses a multi-component approach • Maximises interaction between the different components of the intervention • Addresses systems, operations, structures and relations • Is flexible in meeting the needs of different agencies
Engagement and ownership	• Engages agencies in owning and driving the program • Is tailored to focus on the agency’s priorities
Goal setting and feedback	• Provides feedback about current practice • Provides a clear rationale for change • Develops agreement about concrete and specific change goals • Monitors and provides feedback about change during the intervention program
Interactive skill development	• Provides self-education opportunities and access to resources • Recognises the expertise of participants • Is interactive with a focus on shared reflection and problem solving • Provides opportunity for rehearsal and practice
Leadership, roles and relationships	• Uses champions to model and promote the use of evidence from research (including both internal and external champions) • Uses credible, dynamic experts as presenters

The intervention comprised multiple components: (i) audit, feedback and goal setting; (ii) a leadership program; (iii) organisational support tools; (iv) the opportunity to test systems for accessing research; (v) research access; and (vi) educational symposia. These components had varying degrees of flexibility as outlined in Table 1. Agency staff received approximately 11 face-to-face sessions over the 12-month intervention period, combined with periodic feedback and ongoing access to resources. Proximal and distal outcomes included (1) organisational capacity to use research (staff knowledge, skills and perceptions of the value of research and organisational support for the use of research as demonstrated through leadership support, policies, tools and systems), (2) research engagement (accessing, appraising and generating research, and interacting with researchers), and (3) research use in policy or program work (demonstrated through the assessment of nominated policy documents). Agencies could prioritise outcomes they wished to improve by tailoring the intervention, e.g. to target particular knowledge or skills.

High-profile policy and research experts were recruited to deliver the face-to-face intervention sessions. The outcome measures comprised an online survey and two structured interviews. Further details are provided in the study protocol [66].

### The challenges

Several characteristics of SPIRIT presented challenges for fidelity assessment. Addressing these challenges drove the methods we used:

1. *Composite theory*. The intervention was built on cross-disciplinary composite theory that had not been operationalised in previous trials. This theory was articulated in the SPIRIT action framework and change principles (Fig. 1 and Table 2), but these did not identify which intervention elements should be used as fidelity indicators, nor the level of specificity with which they should be operationalised.
  The manner in which the essential elements should be articulated was complicated by the paradigmatic tensions and different fidelity traditions in the composite theory. For example, cognitive behavioural theories lean towards positivism and experimental intervention approaches and fall within the standardised approach to fidelity assessment outlined at the beginning of this paper in which essential elements are tightly specified. Systems thinking, on the other hand, proposes a complexity-orientated ecological worldview in which interventions are loosely specified for local adaptation and essential elements are articulated as principles rather than concrete techniques. SPIRIT, like many contemporary interventions, was occupying a middle ground.
2. *Flexibility.* The expression of the essential elements needed to accommodate three levels of flexibility: (a) agencies were able to select different session options from a menu of components, (b) they could tailor the topics and goals of these options to address local priorities, and (c) expert providers determined the detail of delivery (see Table 1). We could not foresee how these decisions would shape the content and form of the intervention. Given that meaningful comparison of the extent to which essential elements were delivered required that they be equally applicable across all intervention sites, our fidelity criteria had to cover both standardised and locally adapted intervention components and reconcile potentially disparate adaptions.
3. *Responsivity to context.* The implementation plan was not fully developed when the trial commenced and was going to incorporate a degree of responsivity to shifting agency priorities, so we needed capacity to adjust our fidelity criteria and data collection methods as the need arose. The complexity of the intervention and of the participating organisations precluded any confident prediction about the essential elements’ validity (would they accurately reflect the intervention theory? would they turn out to be essential?) or even their feasibility (could they be implemented as planned?).

## Methods

As a result of these uncertainties, we were unable to predetermine the content, scope and specificity of the essential elements. Consequently, we judged it necessary to identify provisional essential elements and observe them in the field, using empirical evidence from the process evaluation to revise them as required. Our goal was to critique the construct validity of the essential elements [9] and modify them while simultaneously using them as reliable fidelity indicators.

The mixed-method process evaluation focused on three domains: (a) how the intervention was implemented (fidelity assessment), (b) how people participated in and perceived the intervention, and (c) the contexts that mediated this relationship. As shown in Table 3, qualitative and quantitative data collection methods included purposively sampled semi-structured interviews; direct observation and coding of intervention activities; conversations with the intervention designers, implementers and providers; and participant feedback forms. These are described in detail in the SPIRIT process evaluation protocol [67].

**Table 3:** How we answered the three questions for assessing essential elements during the intervention period

Questions used to critique essential elements	Data sources	Data examples	Data analysis / use
1. When implemented in these contexts, does this provisional / likely essential element realise the change principle(s) that informed its development? 2. Is this essential element critical for achieving the session goals? Does anything else appear to be? 3. Does this essential element function across all subcomponents and all six trial intervention settings?	Implementation checklist completed during the delivery of each session	Codes showing whether or not (or to what extent) each essential element was delivered as intended	Collation of codes by session and by agency
	Fieldnotes made during observation of each session	Description of how the essential elements appeared to work or not (e.g. how participants reacted), how they were delivered, any adaptations that took place, any factors that appeared to affect how the intervention was delivered or how people engaged with and responded to it	Data was coded thematically using the constant comparative method. In each session we examined the alignment between 1. what was delivered (including any modifications), 2. any observed process effects, and 3. the change principles that informed what was intended, and compared this across all agencies
	Participant feedback forms collected at the end of each session	How participants assessed delivery against quality criteria such as content relevance, provider credibility, and learning outcomes; and their advice for improvements	Descriptive analysis of quantitative data (frequencies, averages and comparisons)
	Transcripts of semi-structured interviews with purposively sampled participants from two phases of interviewing: early in the intervention period and after it	Participant perceptions of the strategies used to effect change: the extent to which they worked and how modifying factors such as work practices, organisational goals, and beliefs about research shaped process effects	Managed using Framework Analysis. Data was synthesised in categories that were identified both inductively from early interviews and *a priori* based on intervention outcomes and a review of the research utilisation literature
	Fieldnotes documenting informal conversations with participants following sessions	As above but ad hoc and generally very brief	Data was collated in running memos and, where appropriate, coded thematically using the constant comparative method
	Memos documenting conversations with intervention implementers and providers	Implementers’ views on discrepancies between what was intended and what was delivered. Providers’ accounts of why they ‘went off script’	
	Memos documenting consultations with the intervention designers	How the designers envisaged the change principles manifesting in intervention sessions	

The research group (which comprised the intervention designers, implementation team and process evaluation team working in parallel) used the relatively lengthy intervention period as an opportunity to identify, assess and refine hypothesised essential elements during the trial. This was aided by the multi-agency, stepped wedge design of the trial which allowed us to monitor the entire intervention in some agencies and still have scope to trial revisions in other agencies. A modified version of this approach could be applied to other trial designs.

**Fig. 2:**
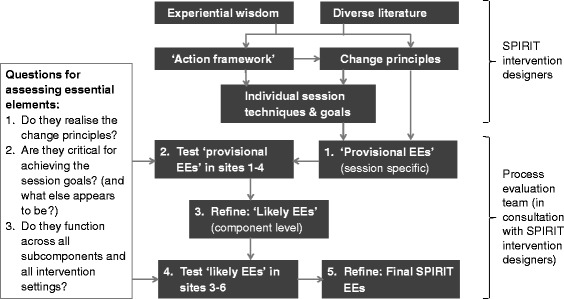
Process for identifying, testing and refining essential elements (EEs)

The provision of a dedicated process evaluation researcher as part of the wider group enabled the collection of multiple forms of evaluative data from all sites, and iterative conversations with the intervention designers about their conceptualisation of the intervention’s causal pathways. This allowed us to assess the validity of the essential elements using a five-stage process. Stage 1: identify provisional essential elements; stage 2: test provisional essential elements in intervention contexts; stage 3: refine provisional essential elements and develop likely essential elements; stage 4: test likely essential elements in intervention contexts; and stage 5: refine the likely essential elements and develop final essential elements. See Fig. 2 for a visual overview of this process. Each of these stages is now described.

## Results

These results overlap with our methods in that we show how process evaluation data collection and analysis was used to critique essential elements. This detail is provided so that the procedure we devised is transparent and replicable.

### Stage 1: identifying provisional essential elements

SPIRIT drew on diverse literature and expertise in its design. As shown in Fig. 2, this body of knowledge was distilled by the intervention designers into an action framework (Fig. 1) and a list of change principles (Table 2) [63, 66, 67] which formed the theoretical basis that we attempted to operationalise in response to each intervention session. These sessions were developed by the intervention designers in consultation with agency staff and expert providers.

We could not use SPIRIT’s change principles as our essential elements. Doing so may have been appropriate for a very flexible intervention with minimally specified, non-standardised components [61]. In such a case, fidelity assessment could focus less on specific operationalisations of the change principles and more on if and how the change principles were realised [59]. However, this was not appropriate for SPIRIT which sought a balance of standardisation and flexibility within a menu of predefined components. The process evaluation aimed to report on variation in the delivery and response to each of these components, consequently the change principles were too abstract to be used as indicators for fidelity reporting. Similarly, the action framework, which functioned as our logic model, outlined causal pathways and relationships in relation to individual and organisational capacity building but did not identify techniques. We needed a concrete and observable expression of what was at the heart of these strategies if we were to identify commonalities and differences in implementation that could help interpret the outcomes and inform further interventions.

The approach we devised was to identify *potential essential elements* inductively. As each session outline became available, the process evaluation team asked three questions. (a) What do the session goals and the planned characteristics of the session tell us about which change principles this session is attempting to utilise? (b) Which of these are likely to be essential to the effectiveness of the session? (c) What would these change principles look like in delivery (how can we operationalise them so that can be measured or fully described?)? This produced a list of draft essential elements that we further developed with the SPIRIT designers to accurately describe the elements they believed were essential for that session to be effective. These potential essential elements included session content, key messages, provider characteristics, presentation techniques, activities, and particular attendees and types of participation. At this stage, we consciously trialled many essential elements that we suspected would be collapsed or discarded later. See Additional file 1 for an example.

Devising potential essential elements also required the operationalisation of some relatively abstract overarching concepts. We describe the development of one of these—the concept of quality—in more detail. This is because it is particularly important for ensuring that intervention objectives are achieved [10], yet is neglected in the literature [12, 68].

As per Dusenbury et al.’s definition of quality as ‘*the extent to which a provider approaches a theoretical ideal in terms of delivering program content*’ ([10]: 244), we conceptualised quality as congruence between (a) the intervention-as-implemented and (b) the intervention theory—in particular, the change principles. The change principles were strongly informed by adult learning theory which provided quality constructs such as: the providers’ content expertise and presentational skills; the extent to which participants found workshops to be interesting, engaging and respectful of their contributions; the relevance and potential usability of the information and ideas provided; and if participants were facilitated to explore how information and ideas might be applied in their work settings [69, 70].

We were able to operationalise some aspects of these quality constructs and so include them as evaluator-coded essential elements (e.g. by devising criteria for ‘content expertise’ and using observations to determine the extent to which information and ideas were discussed in relation to participants’ work). However, because quality is highly situated [12], we considered many aspects would be best assessed by participants themselves. Therefore, items in the participant feedback forms were used to collect information about quality constructs such as content relevance, provider suitability, how engaging the session was and the usefulness of information provided. Quality across the whole program was also considered as part of the semi-structured interviews that were conducted with participants after the intervention. Interviews focused on capturing the breadth of quality criteria from participants’ perspectives (we were mindful that our notion of quality might not align with theirs) and exploring reasons for their judgement rather than ratings.

### Stage 2: testing provisional essential elements in intervention contexts

During the first step of SPIRIT (in which the intervention was fully implemented in two agencies and partially implemented in a further two), the process evaluation team not only monitored adherence to the essential elements but also gathered qualitative and quantitative data that would help us better understand their real world functionality and validity. We conceptualised validity as (1) how well the essential elements embodied and delivered the intervention’s theoretical foundations [6, 9, 71] and (2) the extent to which the essential elements were actually essential in each setting [17] (we were aware that elements which seemed essential in one context might not be so in all contexts and circumstances [13]). Data was collected via observational field notes, checklist coding, post-session memos, participant interviews, participant feedback form ratings and comments, and conversations with providers and implementation team members.

During the concurrent data collection and analysis process, we adopted a stance of ‘naïve curiosity’ in relation to the essential elements, asking ‘What seems to be more or less successful in meeting the goals of each session, and why?’ This enabled us to stay open to potential essential elements that we may have failed to consider prior to the evaluation. For example, we noted early on that participants appeared to engage more with session content and gave more favourable feedback when the provider explicitly recognised the challenges of their work, including having a realistic view of the (limited) role of research within it. When the reverse was observed (participants disengaging because the provider appeared insensitive to this issue), we concluded this concept was an essential element of the relevant components: ‘*Provider demonstrated sensitivity to the ‘real world’ of the agency’s policy/program work*’.

To address our concern about validity we also asked ‘How well was the theory underpinning the intervention realised in the delivery of this session?’ and ‘Does each putative essential element appear to be critical for achieving the session goals?’ Data was synthesised in running memos that identified issues to explore in further sessions. Analysis focused on comparing our data with the program logic and, primarily, with the change principles that had been identified as informing each session plan.

Six (often overlapping) challenges to the validity of the essential elements were identified through this inductive process. Essential elements could be (1) *redundant*—the element was not essential; (2) *poorly articulated*—unclear, too specific or not specific enough; (3) *infeasible*—it was not possible to implement the essential element as intended; (4) *ineffective*—the element did not effectively deliver the change principles; (5) *paradoxical*—counteracting the goals of the session or the underpinning change principles; or (6) *absent or suboptimal*—we identified additional or more effective ways of operationalising the change principles. See Table 4 for examples.

**Table 4:** Challenges to the validity of essential elements for the SPIRIT process evaluation and suggested responses

Challenges: the putative essential element was…	Definition	Essential element example	Comments	Suggested response
Redundant	The strategy described by the element was not essential	‘*The provider encouraged participants to ask questions*’	This was unnecessary in discussion-based sessions where participants interacted as co-contributors	Remove this element
Poorly articulated	The element description was unclear, too specific or not specific enough	‘*The session was introduced by a leader (senior person in the agency* e.g. *CEO, member of executive)*’	This failed to capture the many times that less senior staff introduced sessions that were attended by leaders. This essential element was a proxy for visible endorsement/support (modelling) by organisational leaders which we concluded was also achieved when they attended and contributed enthusiastically to the session in other ways	Hone the description so that it accurately captures the essential element
Infeasible	The essential element described a strategy that was not possible to implement as intended	‘*Participants were facilitated to identify one or more change goals*’	We found this was achievable only in agencies that had developed a research utilisation reform agenda prior to SPIRIT and felt able to use intervention sessions to discuss their goals openly. Other agencies needed more time and different processes to identify goals	Modify or develop alternative strategies. In some cases, the outcomes themselves may need be modified
Ineffective	In practice, the strategy described by the essential element did not effectively deliver the change principles	‘*The provider had experience presenting to policy/program developers*’	This seemed intuitively reasonable as one of several criteria for securing providers with the expertise and credibility stipulated by our change principles, yet there was no correlation between this criterion and our evaluation of session quality or general participant satisfaction feedback	Consider whether this element can simply be removed or if the change principles require further operationalisation to capture an essential aspect of the intervention
Paradoxical	When implemented, the strategy described by the essential element counteracted the session goals or the change principles	No examples of this were identified	Interventions can have counterintuitive impacts. While the process evaluation identified examples of this in other aspects of the trial, none related specifically to the essential elements	Remove this element and consider possible implications for other parts of the intervention
Absent or suboptimal	Additional or more effective ways of operationalising the change principles were identified	‘*The provider persuasively articulated his/her commitment to using research*’*.*	Despite being briefed to do so, many providers did *not* articulate their commitment to using research. However, some used case examples that powerfully illustrated the value of research, and facilitated discussion that enabled participants to express it themselves. This strategy was more sophisticated and a better fit with the adult learning orientated change principles that emphasise interactivity, shared reflection and harnessing participant expertise	Introduce absent elements and modify sub-optimally operationalised elements that the essential aspects of the intervention are captured

Detailed notes were made about the nature of the problem, what interactions affected it (where this was appropriate) and possible solutions that took account of our growing appreciation of contextual constraints and opportunities. Notes included suggestions about where session-specific essential elements could be collapsed and rephrased so that they could be applied across all agencies and intervention components.

### Stage 3: refining the provisional essential elements and developing likely essential elements

The process evaluation team used these notes to amend, distil or reject the 50+ provisional essential elements initially used across the intervention into a list of 26 ‘likely’ essential elements. Following consultation with the intervention designers, these were further revised. The list represented a revised way of articulating and evaluating the fidelity of the intervention but did not affect its design or continuing implementation (with the exception of providers who were sent a list of the essential elements and feedback form items prior to their sessions).

In the revision process, we sought to balance the need for more loosely specified essential elements (which the flexible aspects of the intervention design demanded) with the need to clearly describe what the intervention comprised: not only for the purposes of fidelity assessment but also to provide detailed information that would aid transparent reporting of and potential replication of the intervention. We were guided by Century, Rudnick and Freeman’s account of reducing the granularity with which their essential elements were defined and measured [55]. Consequently, essential elements that had been devised for topic specific sessions were articulated at a higher level of abstraction. For example, ‘*The provider demonstrated the value of using systematic reviews in policy/program decision-making*’ became ‘*The value of using research/evaluation in agency work was conveyed*’. This was necessary because agencies were able to choose and tailor different sessions from within the same intervention component. So in order to monitor fidelity comparatively across all agencies, the essential elements needed to be applicable to every session. Where agencies were able to choose the topic, content, form and goals of face-to-face sessions, the fidelity assessment no longer specified any of these attributes, only that they must reflect the relevant change principles for that component (e.g. those specifying interactivity, shared problem solving, and recognition of participants’ expertise).

### Stage 4: testing ‘likely’ essential elements in intervention contexts

In this stage, we used the likely essential elements in our fidelity assessment data collection and continued using the methods described in stage 2 to collate information about the extent to which they were delivered and to explore their functionality and congruence with the program theory.

### Stage 5: developing final essential elements

Several further changes were made in this stage but, with some exceptions, not as a result of additional information gathered in stage 4. Rather the iterative process of refinement allowed us to reflect on details that had been sidelined by more pressing concerns in the previous stages. Having addressed those, we had capacity to focus on less critical amendments and fine tune some essential elements that might otherwise have been considered ‘good enough’. Our final list of essential elements was reduced to 20 items (Table 5). These included several that we considered collapsing but decided to retain separately. For example, is this provider-related element: ‘*The provider encouraged participants to contribute to session*’ really essential when a participation-related element: ‘*Participants contributed to session*’ addressed the same concept? Based on empirical evidence from the trial, we concluded it was important to differentiate between (and learn from) what was delivered and how people responded. Our observational data showed that in most sessions the providers’ actions appeared to shape the levels and types of participation, but this was not always the case. Also, because providers were given a loosely specified briefing regarding delivery techniques, as befitted the senior experts who were recruited, we felt it helpful to retain the item for instructional purposes.

**Table 5:** Overview of SPIRIT’s final essential elements: their scoring, how they were monitored and which of the interventions components they applied to

Final essential elements^a^		Final scoring of essential element	Activity that provided data for scoring	Intervention components to which essential elements apply			
				Audit & feedback	Leaders forums	Symposia	Research exchanges
1.	Provider had expertise and credentials in the topic/field appropriate to the session	*Yes* / *No*	Review of publicly available biographical information and, for no. 1, participant feedback form item	✓	✓	✓	✓
2.	Provider had experience in presenting to policy/program developers	*Yes* / *No*		✓	✓	✓	✓
**Engagement and facilitation: the methods used to deliver the presentation and encourage participation**							
3.	Non-didactic presentation strategies were used	*Extensive | Moderate | Limited | Not at all*	Direct observation of session delivery	✓	✓	✓	✓
4.	Content was delivered in an engaging manner	*Yes / No*	Participant feedback form item	✓	✓	✓	✓
5.	The provider encouraged participants to contribute to session (ask questions, make comments, provide examples, participate in discussion)	*Extensive | Moderate | Limited | Not at all*	Direct observation of session delivery	✓	✓	✓	✓
6.	The provider encouraged participants to discuss how information / learning from the session might be applied in their setting	*Extensive | Moderate | Limited | Not at all*	Direct observation of session delivery	✓	✓	✓	✓
7.	Provider showed respect for participants’ contributions and work	*Extensive | Moderate | Limited | Not at all*	Direct observation of session delivery	✓	✓	✓	✓
8.	Provider demonstrated sensitivity to the ‘real world’ of the agency’s policy/program work	*Extensive | Moderate | Limited | Not at all*	Direct observation and participant feedback form item	✓	✓	✓	
**Content: key topics, messages, activities and resources**							
9.	Core content outlined in session plan was delivered	Aggregated rating across all items specified in session plan: *Wholly | Mostly | About half | Limited | Not at all*	Direct observation and multiple participant feedback form items	✓	✓	✓	✓
10.	The session content was relevant to the agency’s work	*Yes | No*	Participant feedback form item		✓	✓	✓
11.	Where specified in the session plan, provider identified or provided resources that supported or extended learning from the session	*Yes* / Partially / *No* / N/A *- not specified in plan*	Direct observation of session delivery	✓	✓	✓	✓
12.	The value of using research / evaluation in agency work was conveyed	*Yes | No*	Participant feedback form item	✓	✓	✓	✓
13.	Synthesised data from measures was provided and discussed	*Yes | No*	Direct observation of session delivery	✓			
14.	Opportunities to improve use of research were identified	*Extensive | Moderate | Limited | Not at all*	Direct observation of session delivery	✓	✓		
**Participation: characteristics of attendees’ interaction and contribution to the session**							
15.	Targeted agency staff attended	Numbers and roles of all attendees. Approximate proportion of those targeted	Direct observation and review of data from session ‘sign in sheet’	✓	✓	✓	✓
16.	A leader (e.g. CEO, member of executive) introduced the session or contributed to it positively in other ways	*Yes | No*	Direct observation of session delivery			✓	✓
17.	Participants contributed to session (asked questions, made comments, participated in discussion)	*All | ~ ¾ | ~ ½ | ~ ¼ | Few | None*	Direct observation of session delivery	✓	✓	✓	✓
18.	Participant contributions included knowledge/examples from their own experience	*Extensive | Moderate | Limited | Not at all*	Direct observation of session delivery	✓	✓	✓	✓
19.	Discussion included how information/learning from the session might be applied in their setting	*Extensive | Moderate | Limited | Not at all*	Direct observation of session delivery	✓	✓	✓	✓
20.	Participants identified one or more agency research-related areas that could benefit from improvement	*Yes | No*	Direct observation of session delivery	✓	✓		

^a^Essential elements are one type of fidelity criteria. Other fidelity measures concerning frequency, duration, coverage, etc., plus participants’ perspectives, were collected for each session but are not shown on this table

### Scoring the essential elements

Not all fidelity criteria can be assessed in the same manner [9]. Structural items such as participant attendance and the number, type and duration of sessions are easily observed and can usually be captured numerically. However, process items (which may be more significant in terms of intervention effects [9]) such as presentation styles, types of participation and overall quality tend to be more descriptive and usually require context-sensitive qualitative assessment, especially direct observation [9, 19, 62]. Most of our essential elements were processual so we found that their inclusion in the fidelity assessment required that they be monitored not only in terms of *whether* they were delivered, but the *extent to which* they were delivered and *how* this was done. Our aim was to devise a pragmatic method of standardising observations across sites that could accommodate local adaptation and extensive data collection.

We made three primary adjustments to the scoring as a result of the testing. First, we rejected dichotomised scoring on many items in favour of an ordinal scale. Not surprisingly, we found the *yes*/*no* format we trialled too reductive for the complex processes we were observing. We also trialled several five-point scales (as recommended by Bond et al. [21]) but settled on a four-point descriptive scale of *extensive|moderate|limited|not at all* as providing the necessary breadth and precision for our purposes. The definitions that specified the conditions under which each score was applicable were refined in consultation with the intervention designers and the scale was tested in each agency by two members of the team. All coding was supplemented with descriptive notes.

Second, we developed a scale that could be applied to each customised session (workshop, symposium, etc.) and would thereby enable us to compare session *content* scores across the whole trial. Content was considered to be the aspects of the session that the participating agency had specifically requested. Depending on the nature of the session and the level of detail each agency chose to specify, this content varied tremendously from concrete deliverables (e.g. *an example of a systematic review was provided*) to relatively abstract processes and concepts (e.g. *ethical challenges were explored interactively*). The number of content items also varied from between three to eight. We kept the y*es*/*no* score for each individual item and simply aggregated these using a scale of *wholly|mostly|about half|limited|not at all* for each session. This allowed us to compare the delivery of varied content across all sessions and sites without the requirement for a consistent number of items.

Third, we concluded that we had been unsuccessful in finding semi-objective generalisable ways of scoring certain quality concepts (e.g. Was the presentation engaging? Was the content relevant?). We decided to rely entirely on participant feedback to score these essential elements. See Table 5 for an overview of the final scoring.

We had sufficient data (checklists, descriptive notes, memos and audio recordings) from the intervention implementation in stage 1 to apply these new codes retrospectively to the sessions that informed them.

### ‘Prohibited’ elements

During the trial, we eschewed the concept of ‘prohibited’ [9] or ‘forbidden’ elements [72], but when reviewing the data for stage 5 revisions, we concluded that they could have provided clearer guidance for our providers about the intervention’s underpinning principles. These providers were experts in their field but newcomers to SPIRIT. Despite receiving the essential elements for their sessions in advance, many appeared to apply them selectively. Based on participant feedback and our observations, the following guidance may have helped providers avoid the most common pitfalls:

- *To be avoided*:
  - *Talking down to participants. In particular, failure to recognise their expertise and the complexity of their work.*
  - *Talking* at *participants. Didactic presentations should be interspersed with case examples, activities, discussion,* etc. *Invite questions, ask participants about their views and experiences, and encourage debate.*
  - *Reliance on data/cases from other fields. When information is highly relevant it is more applicable. Where possible, use case examples from the agency’s own work. We can provide assistance with this.*
  - *Squeezing out time for discussion. We conceptualise discussion as a primary mechanism for helping participants integrate new knowledge and think about how it might be applied in their contexts.*

We did not trial this guidance partly because it would have radically changed the provider briefing protocol and partly because of the potential to alienate eminent highly skilled professionals with such censorious (and potentially patronising) guidance. However, we believe that our methods for assessing essential elements, combined with sensitive consultation with the providers, would glean valuable information about the appropriateness and utility of such an approach. Although this paper concentrates on critiquing and revising essential elements in situ as a means of improving validity in novel contextualised trials, where threats to validity can be identified in advance they should be addressed before the intervention is underway.

## Discussion

Identifying an intervention’s essential elements and monitoring them via fidelity assessment is critical for understanding how the intervention worked or why it did not work. Yet, there is uncertainty about how to do this, particularly for novel contextualised interventions (i.e. interventions that blend theories pragmatically and which are designed to be flexible and at least partially responsive to local conditions) [8–10, 20, 55]. How do we determine which elements of such interventions are genuinely essential to their effectiveness? And how do we ensure they are valid indicators of the intervention theory [6, 12, 14]? When attempting to answer these questions we found little practical guidance in the literature and encountered paradigmatic differences and ambiguous terminology. For example, what we call *essential elements* [10, 56] are also known as *essential functions* [59], *essential components* [12], *essential ingredients* [62], *active ingredients* [6, 7, 11], *critical ingredients* [21], *critical components* [55] and *core components* [23, 36]. More importantly, they are not always referring to the same phenomenon and they differ greatly in terms of their relationship to the intervention’s theoretical underpinnings. Some refer theoretically to intervention activities [12], others to theoretical functions [59]; some use the term to include the breadth of fidelity criteria (e.g. intensity and reach) [20], while others limit it to carefully mapped and validated indicators of theory-based models [73] or recommendations [17].

Meanwhile, the perceived value of assessing standardised interventions using universal fidelity criteria is declining. The growth of contextualised interventions mirrors increasing recognition of the complexity of the dynamic real world systems in which they are implemented, and the idiosyncratic and unintended ways that interventions and their context can change one another [41, 49, 59, 74]. The need to figure out what fidelity means in such interventions, and to devise methods for identifying and monitoring elements that are genuinely essential, is more pressing than ever.

In this paper, we describe a novel exploratory incremental test-and-refine process devised to strengthen the validity of a contextualised intervention’s essential elements. This pragmatic approach enabled us to collect fidelity data throughout the trial (despite uncertainty about what the intervention would look like when implemented in each setting), while also assessing how well the intervention’s real world delivery aligned with the theoretical principles that underpinned its design. The literature provides advice for articulating factual, precise and targeted fidelity criteria prior to the intervention e.g. [21] but to ensure our essential elements were valid we needed to attend to the interplay of the intervention theory and design with the intervention settings, providers and participants. This was best done empirically in the context of the trial.

Although we monitored implementation fidelity, our methods focused on understanding the intervention’s theoretical fidelity because, as Hawe argues, ‘*Fidelity resides in the theory of the change process, rather than in any particular technology, component, or delivery channel per se. Thus, the role and meaning behind a particular component, rather than its face value, are what matter*’ ([75]: 313).

Identifying the appropriate level of specificity was a critical aspect of determining the essential elements’ validity. Overarchingly, we moved from a tightly specified approach to one that was more loosely defined, better reflecting the intervention’s scope for expert providers to shape activities, and for tailoring to individual sites. We knew that session-specific essential elements would need to be distilled into higher order items that covered whole components of the intervention, but testing the functionality and theoretical congruence of a wide variety of provisional essential elements in multiple sessions and sites enabled us to explore a breadth of possibilities about what mattered and why, increase our understanding of which intervention elements genuinely appeared to be essential, and experiment with how best to articulate and score them. One outcome of this was to increase the extent to which participant feedback was used to measure quality indicators. This approach accords with calls in fidelity assessment, and in research and evaluation more broadly, to use loosely specified evaluation methods that support local adaptation and which recognise that change processes in complex systems are unpredictable and are often best assessed by those receiving the intervention [7, 38, 58, 59]. While none of the process evaluation data, including the evolving fidelity assessment described in this paper, was fed back into the design or implementation of the intervention during this trial, our approach has potential to contribute formatively to developmental evaluations that shape the intervention during its delivery [52].

Our fidelity data will be analysed in relation to participants’ feedback form ratings for each intervention session. We anticipate that sessions with higher implementation fidelity will receive a higher overall score and more favourable free text responses. It will not be possible to disentangle the implications of fidelity results for individual sessions or components when analysing intervention outcomes as they are thought to function interdependently, but our data will tell us the extent to which the operational and theoretical aspects of the SPIRIT intervention were delivered in each agency. This, in turn, will help us interpret the observed effects of the overall intervention-as-delivered on outcomes.

The use of mixed data collection methods and sources (triangulation), including direct observation and participant feedback, strengthened the rigour of this work [9, 19, 21, 62]. However, the final recursive loop (stages 4 and 5 as described in the ‘Methods’ section) could have been avoided if we had scrutinised all the essential elements with equal emphasis in earlier steps rather than focusing on those with evident problems.

We note that this approach would not be appropriate for all interventions. Given that the modifications mostly either collapsed essential elements or articulated them at a less granular level, we were able to use the data gathered during earlier phases of implementation to apply the modified elements and codes to the sessions that informed them. However, where essential elements are revised to become more granular (as might be the case in standardised programs where highly specified techniques are being honed), our records would not have contained sufficient detail with which to apply codes retrospectively.

There are other limitations. Our lack of independence as members of the wider study team may have affected our ability to observe the intervention implementation dispassionately and, as is always the case, our theoretical and disciplinary allegiances may have skewed what we noticed and how we assessed it. Lastly, what we observed was situational: shaped by the complex interaction between the intervention theory and structure, delivery by multiple providers, diverse participants and distinct organisational contexts, all at particular time points. So, while we believe we have identified elements that are at the heart of the intervention theory, we cannot claim that they will necessarily have equal functionality and validity in all settings and circumstances, particularly where they are expressed with greater specificity [65, 68]. We have, however, honed a list of essential elements that appear to be valid in the context of this trial, and which may provide a starting point for others for interventions similar to SPIRIT.

## Conclusion

This paper describes the difficulties in identifying the essential elements of a contextualised intervention (i.e. an intervention that is informed by composite social and psychological theories and which incorporates standardised and flexible components in order to maximise effectiveness in complex settings). A worked example of an approach for critiquing the validity of essential elements is provided, including a demonstration of how they can be refined during a trial without compromising the fidelity assessment. This process takes intervention evaluators closer to making theoretically and contextually sensitive decisions upon which to base fidelity assessments in trials of contextualised interventions.

## Additional file

Additional file 1:
**Example of how essential elements changed during SPRIT.** (DOCX 30 kb)
