# Supplementary material for: Figuring out fidelity: a worked example of the methods used to identify, critique and revise the essential elements of a contextualised intervention in health policy agencies
Source: Implement Sci. 2016 Feb 24;11:23. doi: 10.1186/s13012-016-0378-6 (PMC4765223; doi:10.1186/s13012-016-0378-6)
Supplement: Additional file 1: — Example of how essential elements changed during SPRIT. (DOCX 30 kb) [file 13012_2016_378_MOESM1_ESM.docx]

**Additional file 1. An example of how the essential elements for the ‘appraising research’ symposium changed during SPRIT**

|  | **‘Provisional’ essential elements**  **(session specific)** | **‘Likely’ essential elements**  **(applicable to all sessions within this component)** | **Final essential elements**  **(applicable to all sessions within this component)** |
| --- | --- | --- | --- |
| **Providers** | The provider had:   - Research credentials at an equivalent level or higher than the attendees - Experience in presenting to policy/program developers | The provider had:   - Expertise and credentials in the topic/field appropriate to the session - Experience in presenting to policy/program developers | The provider had:   - Expertise and credentials in the topic/field appropriate to the session - Experience in presenting to policy/program developers |
| **Facilitation and engagement** | A senior manager in the agency:   - introduced the session - explained that audit had identified a need/desire for this symposium - participated in the session   The presenter:   - acknowledged the sophistication and complexity of policy/program work - acknowledged the challenges and constraints of using research in policy/program work - showed respect for participants’ contributions - encouraged and facilitated: - group discussion - questions from participants - contributions from participants that addressed challenges and/or questions - examples from participants’ practice experience - comments from participants that demonstrated their skills and knowledge - 1 > appraisal examples were worked through in small groups and fed back, OR in larger group with participants providing feedback   Content was delivered in an engaging manner | - A leader (e.g. CEO, member of executive) introduced the session - The provider:   - encouraged participants to ask questions   - encouraged participants to discuss one or more aspects of the topic   - encouraged participants to discuss how the information / learning might be applied in their setting   - showed respect for participants’ contributions   - demonstrated sensitivity to the ‘real world’ of policy/program work - Non-didactic teaching strategies were used - Content was delivered in an engaging manner | - A leader (e.g. CEO, member of executive) introduced the session or contributed to it positively in other ways - The provider:   - encouraged participants to contribute to session (ask questions, comment, provide examples, participate in discussion)   - encouraged participants to discuss how the information / learning might be applied in their setting   - showed respect for participants’ contributions and work   - demonstrated sensitivity to the ‘real world’ of the agency’s policy/program work - Non-didactic teaching strategies were used |
| **Session content** | The presenter:   - described evidence appraisal - persuasively articulated the value of appraising evidence - facilitated discussion about the role that appraisal can & should play in policy/program development, inc acknowledgement of: - time pressures - pragmatism (policy goals may be different to academic goals) - described different types of evidence (e.g. single studies, reviews, meta-reviews, evidence-based recommendations, summaries for policy and practice) - described the value and uses of evidence types, including types of Qs they can answer - used examples to illustrate evidence types - described how to appraise evidence, inc: - what makes a review reliable - assessing applicability: generalisability, relevance, transfer to local settings - described useful evidence sources., inc: - where to find reviews and summaries - what different repositories cover - described useful tools/resources, inc: - where to find info to guide appraisal of individual studies - where to find info to guide own evidence reviews   Content was relevant to the agency’s work | - Core content outlined in session plan was delivered* - The session content was relevant to the agency’s work - Where specified in session plan, the provider identified or provided resources that supported or extended learning from the session - The value of using research/evaluation in agency work was conveyed | - Core content outlined in session plan was delivered - Where specified in the session plan, provider identified or provided resources that supported or extended learning from the session - The value of using research/evaluation in agency work was conveyed |
| **Participation (observed)** | - Targeted agency staff attended - Participants asked questions - Half or more of the participants made a contribution - Participants interacted with each other in discussion or in activities - Participant contributions included knowledge/examples from their own experience - Discussion included how information/learning from the session might be applied in their setting | - Targeted agency staff attended - Participants asked questions - Participants contributed to discussion - Participant contributions included knowledge/examples from their own experience - Discussion included how information/learning from the session might be applied in their setting | - Targeted agency staff attended - Participants contributed to session (asked questions, made comments, participated in discussion) - Participant contributions included knowledge/examples from their own experience - Discussion included how information/learning from the session might be applied in their setting |
| **Participant feedback** | The symposium:   - Increased knowledge about where to find research reviews - Increased understanding of how to assess the quality of research reviews - Successfully made the case that research evidence should be critically appraised for policy/program work - Was relevant to participants’ work - Provided useful information - Was realistic about the challenges and limitations of appraising research for policy/program work   The presenter:   - Was engaging - Had appropriate knowledge and skills   Participants judged it likely that:   - They would use knowledge gained from the symposium in their work - They would participate in further SPIRIT events   SPIRIT will benefit their agency | The symposium:   - Increased knowledge about where to find research reviews - Increased understanding of how to assess the quality of research reviews - Successfully made the case that research evidence should be critically appraised for policy/program work - Was relevant to participants’ work - Provided useful information - Was realistic about the challenges and limitations of appraising research for policy/program work   The presenter:   - Was engaging - Had appropriate knowledge and skills   Participants judged it likely that:   - They would use knowledge gained from the symposium in their work - They would participate in further SPIRIT events   SPIRIT will benefit their agency | The symposium:   - Increased knowledge about where to find research reviews - Increased understanding of how to assess the quality of research reviews - Successfully made the case that research evidence should be critically appraised for policy/program work - Was relevant to participants’ work - Provided useful information - Was realistic about the challenges and limitations of appraising research for policy/program work   The presenter:   - Was engaging - Had appropriate knowledge and skills   Participants judged it likely that:   - They would use knowledge gained from the symposium in their work - They would participate in further SPIRIT events - SPIRIT will benefit their agency |

* The core content for this session comprised four items: 1. What is a systematic review? 2. Where can I find systematic reviews? 3. Methods for appraising systematic reviews, and 4. Practical exercise in which participants work through the appraisal of a systematic review
